# A Community Based Systems Diagram of Obesity Causes

**DOI:** 10.1371/journal.pone.0129683

**Published:** 2015-07-08

**Authors:** Steven Allender, Brynle Owen, Jill Kuhlberg, Janette Lowe, Phoebe Nagorcka-Smith, Jill Whelan, Colin Bell

**Affiliations:** 1 World Health Organization Collaborating Centre for Obesity Prevention, Deakin University, Geelong, Victoria, Australia; 2 George Warren Brown School of Social Work, Washington University in St. Louis, St Louis, Missouri, United States of America; 3 Southern Grampians Glenelg Primary Care Partnership, Hamilton, Victoria, Australia; 4 Portland District Health, Portland, Victoria, Australia; 5 School of Medicine, Deakin University, Geelong, Victoria, Australia; Institute for Health & the Environment, UNITED STATES

## Abstract

**Introduction:**

Application of system thinking to the development, implementation and evaluation of childhood obesity prevention efforts represents the cutting edge of community-based prevention. We report on an approach to developing a system oriented community perspective on the causes of obesity.

**Methods:**

Group model building sessions were conducted in a rural Australian community to address increasing childhood obesity. Stakeholders (n = 12) built a community model that progressed from connection circles to causal loop diagrams using scripts from the system dynamics literature. Participants began this work in identifying change over time in causes and effects of childhood obesity within their community. The initial causal loop diagram was then reviewed and elaborated by 50 community leaders over a full day session.

**Results:**

The process created a causal loop diagram representing community perceptions of determinants and causes of obesity. The causal loop diagram can be broken down into four separate domains; social influences; fast food and junk food; participation in sport; and general physical activity.

**Discussion:**

This causal loop diagram can provide the basis for community led planning of a prevention response that engages with multiple levels of existing settings and systems.

## Introduction

As our understanding of the complexity of obesity causation has matured, system thinking has surfaced as a promising approach to prevention [1–7]. One example of the application of system thinking was the high profile Foresight [8] project where expert views of the complexity of factors affecting energy balance were represented visually using causal loop diagrams (CLD). The resulting ‘obesity systems map’ presents a causal model that begins with energy balance at an individual level and builds a peripheral set of 108 variables that directly or indirectly influence energy balance. Once viewers came to grips with the complexity of the map they started seeing solutions from individual level through to policy settings and all places in between, and therein lies the value of applying systems thinking.

This emerging interest in systems based intervention responds to the growing awareness that the prevention of obesity must engage with the complexity of causes from an individual level through to policy settings. While system thinking has a long history in other scientific disciplines like engineering [9], the application to obesity prevention is recent and not well described. In fact the term ‘systems’ is short hand for a field of intellectual endeavor that could be divided into as many as 50 fields ranging from engineering to anthropology, biology, social sciences and psychology [3,9]. A systems approach takes complexity into account by considering non linear relationships between variables, accumulations, feedback loops, effects of time delays in systems and the unintended consequences that emerge as a function of these characteristics that would otherwise be missed in more reductionist approaches [3].

A logical intersection of the system thinking and current efforts to prevent obesity is community-based interventions. The most recent Cochrane review on obesity prevention suggests that engaging with complexity through multi-strategy, multi-level approaches delivered as community based interventions represents the most promising direction for prevention [10]. To date, successful community based interventions have prioritized process driven approaches which increase community participation, ownership and capacity [11] and apply, either explicitly or implicitly, an iterative design akin to the action research cycle of diagnose, plan, act, evaluate, synthesize and plan [12,13]. It remains a challenge to formally integrate complexity via systems thinking into the phases of community based intervention while still fostering community engagement and ownership.

Within system science approach, system dynamics, there is an established practice of involving stakeholders in the process of building informal maps and formal simulation models called group model building (GMB) [14,15]. Although historically stakeholder participants in GMB have been managers in the private sector or government officials, the potential for including diverse community stakeholders in GMB in order to build their own capacity to understand and address complex public health issues like childhood obesity has been discussed [16]. To date, few examples of community GMB this context have been published.

In this paper we present the results of research conducted with one community as a part of a larger intervention planning process for childhood obesity informed by the following research question:

What kind of consensus and insight can a community gain about the problem of childhood obesity using group model building?

## Methods

Participants provided written informed consent to participate in this study. This study and consent procedure for participants received ethics clearance from the institutional review board of Deakin University. Ethics Committee reference number HEAG-H 155_2014.

We followed the published standard [17] for the reporting of qualitative research under three domains; research team and reflexivity; study design; and data analysis and reporting.

### Research team and reflexivity

A ‘core modeling team’ (SA, BO, JK, JL, PNS, JW, CB) worked with a project steering committee to define goals of the research, pilot methods and collect data over each step in the process. Research protocols were developed using GMB scripts and “scriptsmap” [16,18,19]. These scripts describe the essential components of an exercise along with the inputs from other exercises needed to do the script and the outputs produced from the script [15,20]. The core modeling team convened a facilitation team that drew experts from local government and health services, and professionals with expertise in GMB, system dynamics and obesity prevention. The range of roles included meeting opener/closer, modeler, facilitator, recorder, wall builder, reflector, production coordinator and debriefer [16]. All team members received training in both GMB methods and large and small group facilitation.

### Study design

This research was developed from a system dynamic theoretical frame stance which aims to underpin the mechanisms driving dynamic behavior by identifying causal relationships, feedback loops, delays and unintended consequences [16]. Data collection occurred in two phases: key stakeholder consultation and broader community consultation.

### Key stakeholder consultation

The aim of the first phase was to conduct GMB to integrate knowledge and experience of stakeholders from different sectors about the system drivers of childhood obesity in their community.

Participants were key informants identified as community leaders whose roles directly impacted on pre-adolescent environments. Workers from the local health service, local government or Primary Care Partnership approached potential participants. Of the 13 identified in the initial review of potential participants, 12 participated. The roles of participants in the community are described in Table 1.

**Table 1:** Participant titles and roles.

Title	Role
Chief Executive Officer (CEO), Primary Care Partnership	Coordinates regional responses to health issues, brokers partnerships
CEO District Health Service	Manages operations of local acute and sub-acute public health care service
Director of Primary and Community Health, District Health Service	Manages sub-acute services provided to community e.g. allied health, health promotion and district nursing
Healthy Lifestyles/Tobacco Action Worker, Aboriginal Health Clinic	Runs health promotion programs targeting local Aboriginals, particularly chronic disease prevention
CEO, Disability Service	Manages provider of respite care, adult education and activity programs for local people with disabilities
Board Member District Health Service/Local Government Councilor/ Executive Officer Local Business Association	Member setting strategic direction and governance for District Health Service and Local Government Area. Manages Business Development Association
Youth Development Officer, Local Government	Works to increase youth engagement with local issues and participation in problem solving; coordinating youth activities
Active Communities Manager, Local Government	Coordinates the implementation of Municipal Public Health and Wellbeing Plan, manages recreational facilities and community development programs
Maternal and Child Health Coordinator, Local Government	Coordinates Maternal and Child Health Services including clinics and engaging vulnerable families
Health Promotion/Community Development Workers, District Health Service and Local Government	Works to increase capacity of local community to maintain good health and address emerging health issues
Children's Services Coordinator, Local Government	Coordinates local early years services including child care and kindergartens

Setting: Data were collected over two 90-minute breakfast meetings, held one week apart, in a mutually convenient restaurant location. The setting represents a rural area which relies heavily on agriculture, marine production and heavy industry including power generation and steel production, The population consists of a majority white Anglo-Saxon of European heritage and a strong indigenous community.

Data collection: Session 1—Participants received an overview of the project and were then asked to consider obesity as a dynamic problem and identify as many variables as they could that have affected or been affected by obesity in this community over time. The community were presented with a graph of changing childhood obesity prevalence over time and asked to create their own graphs of other changes that have occurred over a similar time period affecting or affected by the trend observed in childhood obesity (known as graphs over time) [16,18]. During group discussion of these lists the modeler recorded each variable in the VenSim, [21] which is a software package used routinely to build causal loop diagrams and which allowed for the emerging model to be projected on a wall. Participants identified connections [18], causality and the nature of the relationship between variables. The nature of each relationship was described as either positive or negative (i.e. a positive relationship refers to one where variables change in the same direction and a negative relationship where they change in opposite directions). Participants were encouraged to add or refine variables and review connections continuously. The note taker recorded conversations verbatim as the modeler developed the connection circle in real time. Between sessions the connection circles were developed into a causal loop diagram (CLD). The two members of the core modeling team who had acted as the modeler and recorder reviewed the group session notes alongside the initial model, verifying that all of the connections discussed were accurately reflected in the diagram. Variable clusters were identified and organized according to the emergent themes by members of the core modeling team. Any changes made to the variable names or linkages generated at the first session were reflected in a separate color and flagged for discussion at the following GMB session.

Session 2 –key stakeholders reconvened and were reintroduced to the project aims and reminded of the connection circle created in session one. The process of refinement to a CLD was described to introduce the current CLD. Participants reviewed the CLD, added any other variables or connections to the diagram, and corrected any errors or misrepresentations in the way their stories were reflected in the diagram generated the week before. Note takers recorded the stories being shared and any negotiations around the meaning of variables. In each of the steps described above data collection continued until the number of ideas began to recede and a point approaching idea saturation appeared to have been reached.

Following this session the model was further refined to support a larger community consultation. The modeler and recorder analysed the group session notes alongside the model. The identified variable clusters were modified, updated and organized according to emergent themes. Variable names, direction of linkages, polarity (association/direction) and themes were discussed and debated by the research team to arrive at consensus around best reflecting the perspectives of the stakeholders in their language.

### Broader community consultation

Participants: Potential participants were identified by a working group, which comprised members of local government and health organizations. The working group invited community members with a key role with or significant influence on pre-adolescents or their environments (e.g. education, recreation, sporting, health and business settings). Participants were approached face-to-face, using email and by telephone. A standard script, developed by the working group, was used to engage participants and modified based on the existing relationships with participants.

This process led to 49 participants across the different sessions. A further 43 people were approached who did not participate; 18 cited work commitments, 10 did not return phone calls or emails at all, 8 stated that they weren't interested or did not see applicability to their role, 3 had engaged but did not attend and 4 were interested but away from Portland during the workshops. Several community members sent representatives to the workshop they could not attend.

Participants had in common influence over a setting or environment used by pre-adolescents, but represented a variety of interests and priorities including local planning decisions, sports club participation, providing and/or selling food to children, student wellbeing and business viability.

Setting: Data were collected during a full day community meeting(s) held in a large community space with requisite wall space, audiovisual requirements, etc.

Data collection: Participants were divided into five groups of 12, each with large (1.8m x 1.8m) pictures of the CLDs on the walls. The project purpose and aims were introduced along with conventions of CLD, notably casual direction, polarity and feedback. An expanded core modeling team (<<initials removed for blind review>>) worked with each group to familiarize them with the CLD maps and how they can be understood. Participants placed feedback directly onto the CLD maps using sticky labels (green for agree or support elements in CLD, yellow for unsure and red for dislike or disagree with elements in the CLD) and the research team recorded the placement and content of notes using photographs for later analysis. Note takers were placed with each of the five groups to capture discussions via field notes. In each of the steps described above data collection continued until the number of ideas began to recede and a point approaching data saturation appeared to have been reached.

The facilitator and recorder revised and updated the CLD based on group notes and sticky labels from this session.

## Results

### First key stakeholder consultation

**Fig 1:**
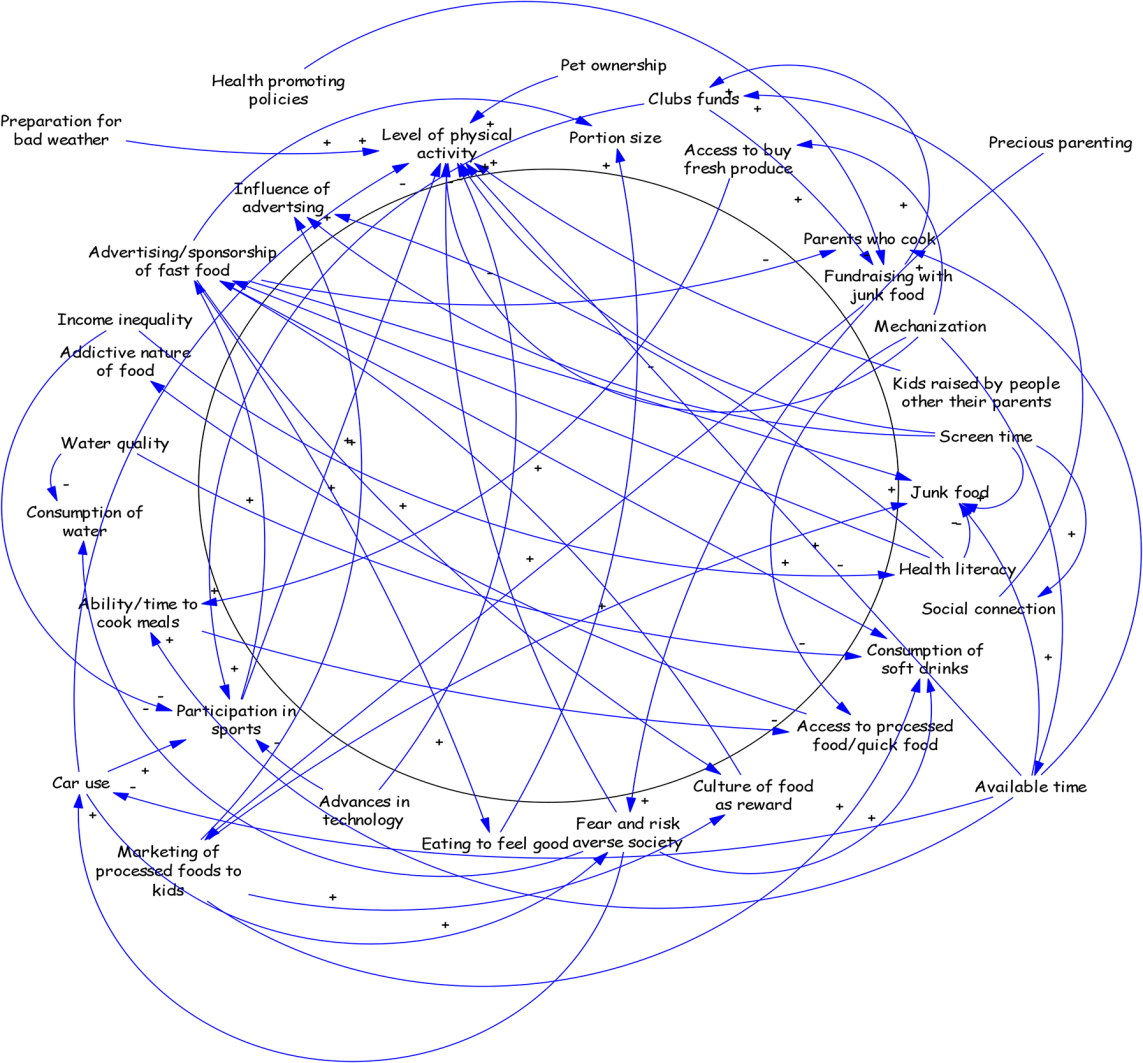
Connection circles for community causes of childhood obesity.

Via the initial connection circle (Fig 1) participants identified multiple levels of determinants (e.g. individual consumption of sugar sweetened beverages) to environmental determinants (e.g. water quality) and policy settings (e.g. health promoting policies) and connections between each. Selected variables and connections are described in detail below.

Field notes indicated identification of connections between physical activity levels and the effect of marketing on children further led to discussion on the contribution of screen time. A cluster of variables describing social connection were identified as related to screen time. This was discussed in the context of reduced social activity within service clubs and community organizations. The double-edged sword of “advancement in technology” (defined as an all-encompassing term for the access and use of automobiles as well as farming technologies that reduced the hours of work hours on farms) was discussed as it on one hand facilitated children’s participation in sports, for example by giving them access to transportation to arrive at practices and games, but on the other hand reduced physical activity levels, for example, technology has reduced the intensity of physical activity associated with farm work. Sports participation was linked to fast food consumption through sporting images in fast food television advertising but also through logos on uniforms and club facilities due to sponsorship.

Consumption of soft drink was described in the context of water quality. Poor tasting municipal water taste was seen as a reason for children choosing sugar sweetened beverages in preference to water from the tap. Moreover, bottled water was perceived as more expensive than bottled sugar sweetened beverages (called ‘consumption of soft drink’ in Fig 1).

### Second key stakeholder consultation

**Fig 2:**
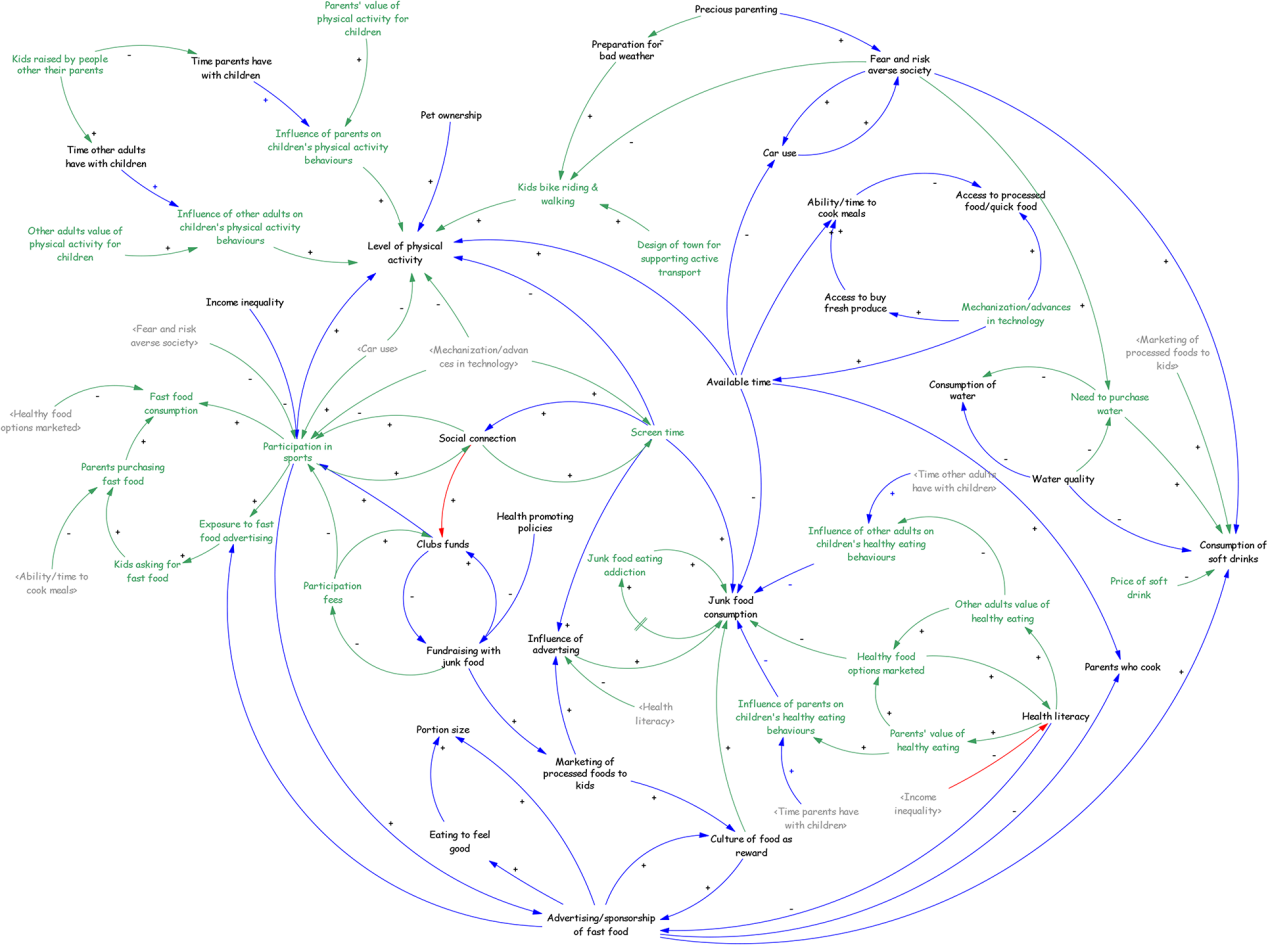
Connection circle to causal loop diagram for community causes of childhood obesity.

The second consultation involved reviewing and refining the causal loop diagram developed from the connection circle (Fig 2). Participants immediately began to identify characteristics of their community that were contributing to childhood obesity (for example fast food, low sport participation and poor water quality). Overall participants appreciated having a visual representation of the obesity problem because it highlighted the complexity but was clean and easy to follow, linkages were clear and it acknowledged and displayed the whole story. Participants very quickly moved to suggest there were gaps that needed to be filled and concepts that needed more consideration. In other words, they started identifying solutions.

While working with the CLD maps participants began to identify reinforcing feedback loops (dubbed ‘whirlpools’ by some participants) and delays. For example, one participant described how social connection had been added as a whirlpool and that screen time was part of the social connection feedback loop leading to reduced activity. The participants then placed this in dynamic context by describing that traditionally social connection would have had the opposite effect of increasing activity through participation in community activity.

The group identified two other reinforcing feedback loops related to dietary behaviors. One demonstrated how eating more fast food over time can create addiction and the addiction increases the amount of junk food consumed. Also, as parents’ ability to cook increases, the number of parents who cook increases. The group agreed this could not happen quickly but over time, given the appropriate training and resources, cooking skills could increase leading to more frequent home cooked meals for many families. Another reinforcing loop was identified as the relationship between physical activity norms and physical activity in children; as more children engage in physical activity, it is perceived as more normal, thus encouraging more children to be engaged in physical activity.

Stakeholders also discussed how poor water taste increased the consumption of soft drinks, and increased soft drink consumption decreased water consumption, which is currently operating as a negative reinforcing feedback loop due to historically poor water taste in the community.

Two important balancing feedback loops involving participation in sport were also discussed in the group. The money required to fund sport clubs can currently come from two different sources, participation fees or fundraising. While fundraising keeps participation fees low so that more children can have the opportunity to join, they were able to identify that it has the unintended consequence of exposing children to more fast food marketing and junk food (as the sale of these is a form of fundraising).

Gaps identified by participants in the first iteration of the map included education and participation in school, and the role of curriculum in supporting physical activity and normalizing healthy eating. The school and education discussion ranged from positive effects to negative effects including the provision of information without necessarily increasing health literacy. Participants felt the model had captured the issues surrounding vulnerable families, particularly drug use, alcohol consumption and issues facing Indigenous communities.

### Large community consultation

**Fig 3:**
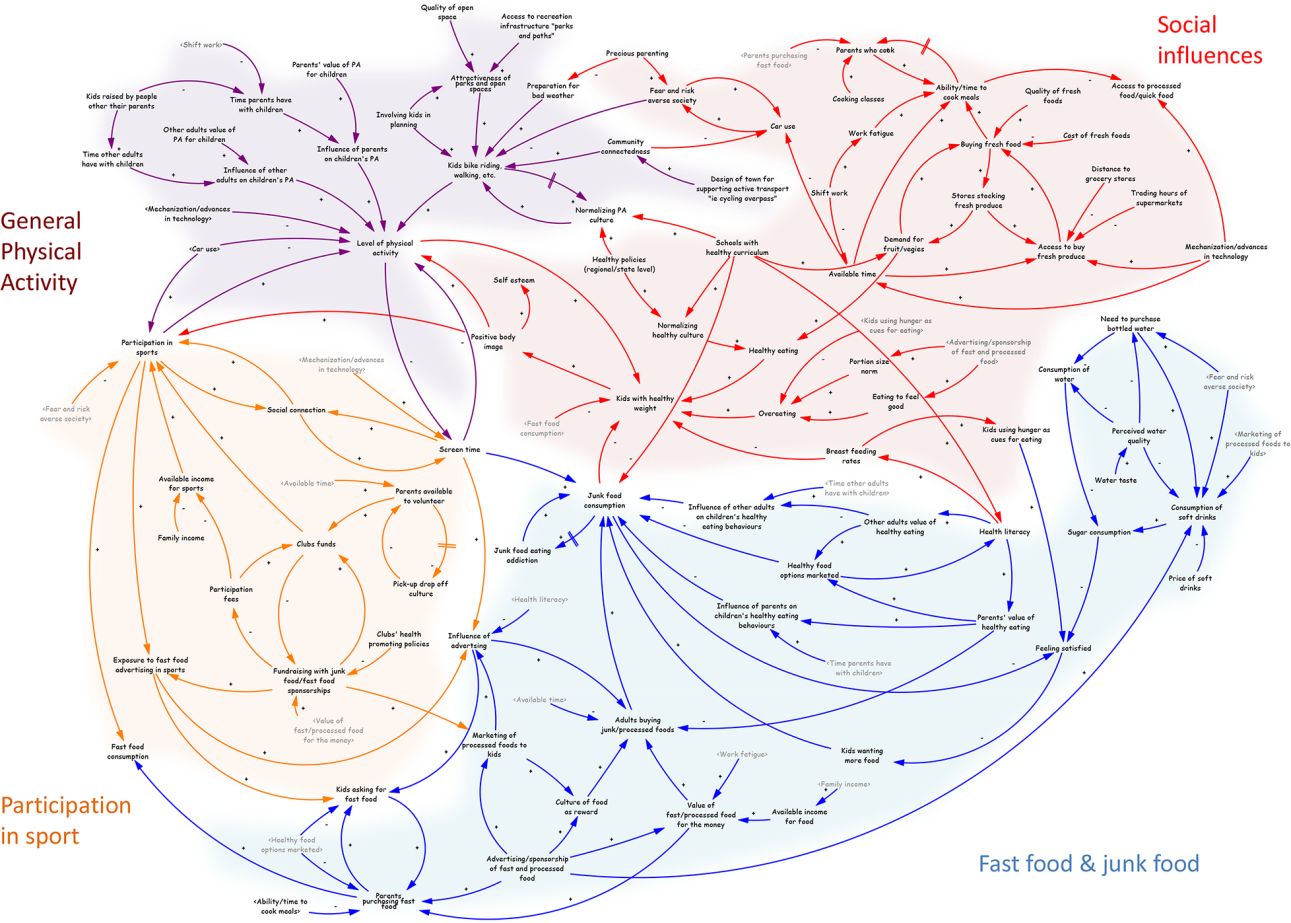
Causal loop diagram of cause of childhood obesity in community.

The first full causal loop diagram (Fig 3) was presented to a broader group of 44 community leaders, with four different domains highlighted: social influences, fast food and junk food, participation in sport and general physical activity. Participants took some time to learn to read the diagrams and then transitioned to providing feedback as to how the diagrams could be improved. Initial gaps identified included medication and relationships between mental illness, motivation, physical activity, and unemployment.

Positive feedback confirmed variable names and links between variables and clusters across the five working groups. For example, all five groups formed clusters of green notes with ticks of approval regarding variable names and relationships across each domain. Neutral feedback was randomly dispersed across all groups and was common when participants were unsure of link between a variable to the problem of childhood obesity. The majority of negative feedback across all groups was focused on general physical activity and participation in sport. All five groups identified variables missing within these clusters and added comments to fill the perceived gaps. The dominant theme was identified as club culture whereby cliques within clubs acted as a barrier to participation in sport and therefore physical activity. A second barrier was a perceived macho culture of the clubs centered on heavy alcohol consumption and risk taking behavior, resulting in parents being apprehensive about allowing their child to participate in sports club activities.

## Discussion

### Statement of principal findings

It was possible to generate a pictorial representation of one community's understanding of the systemic causes of childhood obesity using a causal loop diagram. Participants were able to identify and explain determinants in line with systems thinking, identifying positive and negative feedback loops and time delays. The group model building process and iterations of models produced during the sessions facilitated deeper discussions about the complexity of childhood obesity in Portland and a coordinated response to it that would have been more difficult without a model.

### Strengths and weaknesses of the study

If systems thinking is to become part of normal community intervention practice then a standardized process, as presented here, for the formative stages of intervention development will be critical [11]. This study demonstrates a robust, systematic process to engage community members in developing an understanding of systems that affect childhood obesity that may be generalizable across communities. An additional strength was the ability of this process to develop consensus at each of the stages of community consultation while acknowledging and maintaining areas of disagreement. The approach also explicitly and deliberately engages the community in all steps of the process and provided the basis for subsequent interventions to be owned and driven by that community. The approach was participatory and intended to provide experiential learning of the basic techniques and concepts underpinning this approach to considering systems. This was demonstrated whereby during each iterative step participants picked up a deeper understanding of the picture relatively quickly and moved to making suggestions. A related strength was that the approach engaged people at various levels in the community from leaders to those involved in service delivery. This has the potential to enhance the community readiness for change because the political will, leadership, and workforce are all on the same page and ready to go. A further strength was the development of community capacity to apply systems thinking, with the facilitation team including community members from the start whose roles in the facilitation, analysis and reporting of results became more and more central over subsequent phases. The success of this was in no small part due to the facilitation manuals derived from Scriptapedia [18] which represent tried and tested processes, including facilitation techniques, and so provide materials to develop capacity within teams.

The diagrams provided a useful representation of a system but it would be limiting if it were considered a final model. The map should be dynamic and respond to the changing nature of the problem and evolve to become more solution oriented. A limitation to the approach described here is that it requires a large number of staff and a long lead and preparation time to work with a relatively small number of community members. One corollary of this is difficulty in ensuring all views in the community have been heard; the process is therefore not representative of the full community view. A third limitation may be that the diagrams produced are not generalizable to the broader population or perhaps even the specific community where the work was conducted as it may be too dependent on who is in the room when the map is developed. While these are reasonable critiques the purpose here is to provide a means by which community members can begin to engage with, and apply, systems thinking. At this formative stage of intervention design the process would appear to have achieved this aim.

### Strengths and weaknesses in relation to other studies, discussing particularly any differences in results

This CLD map is relatively complex, however visualizing complexity is part of the aim of the exercise and it is in line with other global models of complexity applying systems based approaches to understand obesity. While this has been useful in describing the complexity of obesity, criticism includes the tangled nature of this ‘spaghetti map’ and some have sought to try and reduce the elements to fewer important variables [22]. A critique of Foresight is that it was expert led, or top down, which contradicts the edict for community based approaches to generate community capacity and ownership. The current approach provides a similar picture of complexity but one which is grounded strongly in, and developed and owned by, the specific community that includes leaders along with other community members [23]. This grounded approach highlighted some important contextual differences between the participants' community and academic models, notably these participants identified water quality and sports program subsidies as important in their context though these issues are not addressed directly within the Foresight model.

### Meaning of the study: possible mechanisms and implications for clinicians or policymakers

This provides a technique policy makers could use to engage communities in systems thinking and may provide the basis for the planning of community-based interventions applying a whole of system approach. It also provides a technique to garner communities' perceptions of the causes of obesity at the same time as developing a shared understanding of the inherent complexity across the multiple determinants. For clinicians it has the potential to provide community based activities that parents concerned about the weight of their child can participate in.

### Unanswered questions and future research

This process has provided one insight into the ways in which a community views the complex determinants of obesity. Further work is required to examine whether this makes an effective starting point for developing whole of system community based intervention. This also raises issues about the ways in which an initiative with this heritage may be evaluated. If the community system is the target for intervention and this takes the form of a process over a package [24] then traditional randomized control trial methods are likely to be less effective. A large body of work is required to apply the range of analytical tools available for analysis systems in public health, which include the four broad categories of system dynamics, social network analysis, agent based models and system identification modeling [3].

Additional work and broader consultation may provide the means to develop a more representative map of system determinants. Developing data driven models on the basis of these initial pictures could also generate further insight into the applicability and utility of the initial model. A useful framing of the different levels of system insight is given by Hovmand [16]. The applications of this approach range from; basic diagrams creating awareness that there is a system and that complexity is an important consideration; to graphical models and maps showing that a system is comprised of multiple components and underlying structures; to identifying leverage points for change within a system; to mathematical models to simulating behavior of a system and helping to explain why things happen [25].
